# Genomic Epidemiology and Machine Learning–Based Drug Discovery for Antimicrobial Resistant Diarrheagenic *Escherichia coli*

**DOI:** 10.1002/mbo3.70236

**Published:** 2026-02-22

**Authors:** Ayesha Masood, Fatima Noor, Abdu Rehman, Mohsin Gulzar Barq, Shazia Iqbal, Muhammad Qasim Ali, Shahzad Ahmad, Syed Zeeshan Haider Naqvi

**Affiliations:** ^1^ Institute of Molecular Biology and Biotechnology (IMBB) The University of Lahore Lahore Pakistan; ^2^ Department of Pathology University College of Medicine and Dentistry, The University of Lahore Lahore Pakistan; ^3^ Center of Bioinformatics, College of Life Sciences Northwest Agriculture and Forestry University Yangling Shaanxi China; ^4^ Department of Microbiology University of Central Punjab (UCP) Lahore Pakistan; ^5^ Faculty of Medicine and Health Sciences The University of Buckingham Buckingham UK; ^6^ Microbiology Department of Gulab Devi Educational Complex Lahore Pakistan

**Keywords:** diarrheagenic *Escherichia coli*, machine learning, molecular docking, molecular dynamic simulation, resistance

## Abstract

Diarrheagenic *Escherichia coli* (DEC) is a leading cause of pediatric diarrhea, with antimicrobial resistance (AMR) complicating treatment. This study analyzed 350 *E. coli* isolates (175 DEC and 175 non‐DEC) to determine molecular pathotypes, resistance patterns, and therapeutic targets. Polymerase chain reaction and 16S ribosomal RNA sequencing identified enteropathogenic *E. coli* as the most prevalent DEC pathotype (35%), followed by enterotoxigenic *E. coli* (25%), enterohemorrhagic *E. coli* (15%), enteroinvasive *E. coli* (10%), and diffusely adherent *E. coli* (20%). Phylogenetic analysis confirmed distinct clustering between DEC and non‐DEC strains, revealing their evolutionary relationships. Antimicrobial susceptibility testing showed high resistance to ampicillin (87.6%), trimethoprim‐sulfamethoxazole (75.5%), and erythromycin (100%), while carbapenems and colistin retained effectiveness. Functional analysis using phylogenetic investigation of communities by reconstruction of unobserved states (PICRUSt) indicated enhanced metabolic and immune‐related functions in DEC strains, differentiating them from non‐DEC strains. Machine learning and bioinformatics‐driven drug discovery identified Alatamide and Isosativan as potential therapeutic compounds, exhibiting strong binding affinities and structural stability against DEC virulence targets through molecular docking and molecular dynamics simulations. This study provides critical insights into the epidemiology, genetic diversity, and resistance patterns of DEC and non‐DEC strains. The integration of bioinformatics and machine learning offers a promising strategy for discovering alternative treatments. Continuous AMR surveillance, responsible antibiotic use, and further experimental validation of identified drug candidates are essential to managing *E. coli*‐associated diarrheal infections in pediatric populations and mitigating the global burden of multidrug‐resistant pathogens.

## Graphical abstract

This study integrates genomic epidemiology and machine learning–based virtual screening to identify novel therapeutic candidates, Alatamide and Isosativan, with strong binding stability against multidrug‐resistant diarrheagenic *Escherichia coli* virulence targets, highlighting computational drug discovery as a promising strategy to combat antimicrobial resistance in pediatric diarrheal infections.

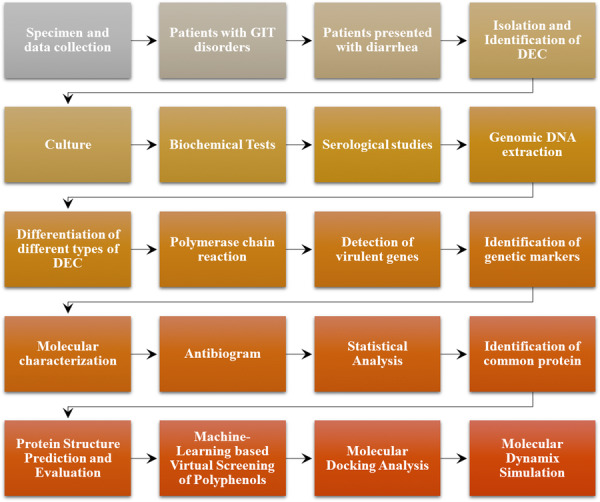

## 1 Introduction

Diarrheal diseases remain one of the greatest global health threats, impacting children the most in low‐ and middle‐income countries (Manetu et al. 2021). *Escherichia coli* is one of the most important bacterial pathogens in the infectious diarrhea syndromes (Robins‐Browne and Hartland 2002). *E. coli* can be a commensal organism of the human gut, but some strains are able to acquire virulence factors and cause diseases of the gut and other organs (Shumi Gebisa 2019). The pathogenic strains of *E. coli* are referred to as diarrheagenic *E. coli* (DEC) and include the pathotypes enteropathogenic *E. coli* (EPEC), enterotoxigenic *E. coli* (ETEC), enterohemorrhagic *E. coli* (EHEC), enteroinvasive *E. coli* (EIEC), enteroaggregative *E. coli* (EAEC), and diffusely adherent *E. coli* (DAEC) (Hassan et al. 2021). Each of these pathotypes as a result of the varied clinical manifestations of the diseases, which can include complications as severe as the EHEC‐associated hemolytic uremic syndrome (HUS), has a unique mechanism of pathogenesis (Goldwater and Bettelheim 2012).

Pathogenesis of DEC is largely driven by specific virulence genes that encode toxins, adhesins, and secretion systems responsible for colonization and host cell damage. For instance, EPEC carries the *eae* gene encoding intimin, which mediates attachment and effacement lesions on intestinal epithelial cells. ETEC expresses heat‐labile (*elt*) and heat‐stable (*est*) enterotoxins that stimulate excessive fluid secretion, leading to watery diarrhea. EHEC possesses *stx1* and *stx2* genes encoding Shiga toxins that can cause severe complications, such as HUS. EIEC harbors *ipaH* and *ial* genes facilitating epithelial invasion similar to *Shigella* species, while EAEC expresses *aggR*, *aap*, and *AAF* fimbriae involved in biofilm formation and persistent colonization. DAEC contains the *daaE* gene, enabling diffuse adherence to epithelial surfaces. Collectively, these virulence determinants underpin the molecular diversity and pathogenic potential of *E. coli* strains, influencing disease severity and clinical outcomes.

Understanding the scope of diarrhea‐associated *E. coli* and particularly, DEC infections is valuable to comprehend the geographic range of the organism (Williams et al. 2012). Due to poor sanitation, inadequate healthcare, and limited access to clean water, countries such as Pakistan focus on the public health impact. Worldwide, *E. coli* is responsible for 30%–40% of diarrheal diseases, with DEC strains contributing significantly to the public health problem (Mohamed and Habib 2023). However, there remains a substantial lack of epidemiological data regarding the prevalence, distribution, and molecular characteristics of these strains, as well as nondiarrheagenic *E. coli* (non‐DEC) strains linked to specific geographic areas, such as Pakistan. The understanding of the circulation of *E. coli* strains in Pakistani children is further hampered by the lack of data pertaining to virulence factors, antibiotic resistance, and outbreak reports at the subnational (district) level (Riaz et al. 2023). Primary data are essential in the planning of well‐defined prevention, control, and treatment strategies, as they provide a basis for the design of elements in these strategies.

Antimicrobial resistance (AMR) poses a considerable challenge when trying to manage infections due to resistant strains of the diarrheal pathogen DEC. The growing number of multidrug‐resistant (MDR) (Alara and Alara 2024) *E. coli* infections, mainly due to the misuse of antibiotics, has jeopardized treatment options and increased the likelihood of more serious health complications. The DEC strains are resistant to some of the most common antibiotics, such as β‐lactams, sulfonamides, fluoroquinolones, and macrolides (Scott et al. 2016). The rise of *E. coli* strains that are resistant to carbapenems creates a substantial need for action to manage resistance of diarrheal pathogens. The rise of AMR justifies the need for more studies to develop new antimicrobials and for more studies to determine the extent of resistance.

Molecular methods, such as polymerase chain reaction (PCR), 16S ribosomal RNA (rRNA) gene sequencing, and phylogenetic analyses, are some of the ways to explain the genetic and evolutionary differences of DEC strains and non‐DEC strains. These methods also outline the particular virulence factors of certain pathotypes, which helps clarify the pathogenicity, as well as the propagation potential of the respective strains. The new technical methods, such as computational molecular docking and more general AI methods, can provide new opportunities for developing specific anti‐DEC virulence factor therapies.

Within this research, studies include investigations involving the prevalence, molecular composition, and AMR of DEC and non‐DEC strains from pediatric patients who present with and without diarrhea. Looking to determine what strains of *E. coli* are diarrheagenic or nondiarrheagenic, assess the major virulence factor genes, and evaluate the resistance patterns to different antibiotics, the research uses 350 *E. coli* isolates from stool samples and rectal swabs (Adesola and Moses 2022). In addition to this, research in DEC treatment alternative therapies uses computational drug discovery methods. This research will likely address the treatment of DEC infections, assist in public health research, and address the problem of MDR *E. coli*.

## 2 Materials and Methods

### 2.1 Study Design and Sample Collection

This study was conducted to isolate, identify, and characterize *E. coli* strains from pediatric patients presenting with diarrheal and nondiarrheal conditions. Stool samples were collected from 350 pediatric patients aged 0–5 years at Mayo Hospital Lahore and transported to the microbiology laboratory for processing.

Inclusion criteria included children aged 0–5 years who presented with diarrhea (for DEC group) or without gastrointestinal symptoms (for non‐DEC group) and whose parents or guardians provided informed consent. Exclusion criteria were children who had received antibiotics within the past 2 weeks, those with chronic gastrointestinal disorders, or incomplete clinical data. To minimize selection bias, samples were collected consecutively from both diarrheal and nondiarrheal pediatric patients during the same study period, ensuring comparable recruitment from similar hospital wards and demographic backgrounds. This study is approved under reference number CRiMM/22/Research/147.

### 2.2 Isolation and Identification of *E. coli*

Stool samples were cultured on MacConkey agar and eosin methylene blue (EMB) agar under sterile conditions. Lactose‐fermenting colonies with characteristic metallic green sheen on EMB agar were presumed to be *E. coli*. Biochemical tests, including the indole test, methyl red test, Voges–Proskauer test, and citrate utilization test, were performed for confirmation. Identified isolates were stored at –80°C in tryptic soy broth containing 15% glycerol for further analysis (Tiemtoré et al. 2022). All isolates were confirmed as *E. coli* based on Gram staining, morphological, and biochemical characteristics.

### 2.3 Antibiotic Susceptibility Testing

The methodology employed in the study is thoroughly planned by Clinical and Laboratory Standards Institute (CLSI) (Hounkpe et al. 2023). The patterns of antibiotic resistance in the isolates were determined using the Kirby–Bauer disk diffusion method on Mueller–Hinton agar, as well as the API 20 NE system for ampicillin, ceftriaxone, ciprofloxacin, trimethoprim/sulfamethoxazole, and several other antibiotics. In the context of this study, MDR was considered to pertain to resistance against three or more antibiotic classes. The CLSI guidelines enabled the assignment of antibiotic susceptibility patterns, thus facilitating the resistance determination ascertained for the different pathotypes of *E. coli*. The isolates were then organized by the type of resistance those mechanisms contained.

### 2.4 Molecular Characterization

For confirmed *E. coli* isolates, genomic DNA was extracted using a commercial bacterial genomic DNA extraction kit. The extracted DNA was also characterized using molecular methods. Specifically, the presence of important virulence genes was detected using PCR. In the case of diarrheagenic strains, pathogenic genes were further characterized using gel electrophoresis. The following virulence genes were screened to differentiate major DEC pathotypes: *eae* (EPEC), *elt* and *estA* (ETEC), *stx1* and *stx2* (EHEC), *ipaH* and *ial* (EIEC), *aggR*, *aap*, and *AAF* (EAEC), and *daaE* (DAEC). The presence or absence of these genes was used to categorize isolates into respective pathotypes.

### 2.5 Comparison of DEC and non‐DEC

Molecular profiles of the isolates were compared between diarrheagenic and nondiarrheagenic groups. Multiple sequence alignment of amplified gene sequences was performed using Clustal Omega to identify mutations and variations associated with pathogenicity. Genomic DNA extracted from isolates showed distinct, clear bands on agarose gel electrophoresis, confirming successful DNA isolation. Amplified products of the 16S rRNA gene confirmed *E. coli* identity at the molecular level. The sequencing results matched *E. coli* reference sequences in the NCBI database, validating species‐level identification.

### 2.6 Antibiogram Comparison

Statistical analyses were conducted to compare the resistance patterns of the antibiograms of both diarrheagenic and nondiarrheagenic isolates. The determination of statistical significance was conducted using the chi‐square and Fisher's exact tests as appropriate.

### 2.7 Bioinformatics Analysis

Pathogenic gene sequences were assessed for therapeutic potential using multiple sequence alignment and mutational analysis. The therapeutic candidate compounds were predicted using machine learning–based virtual screening. Interactions of the predicted candidate compounds with the target proteins were assessed through molecular docking and molecular dynamics (MDs) simulations to determine binding affinity as well as the stability and efficacy of the complexes.

### 2.8 Machine Learning Models Implementation

The classification of active and inactive compounds was done through machine learning algorithms, such as Support Vector Machine (SVM), *k*‐Nearest Neighbors (kNNs), Naive Bayes (NB), and Random Forest (RF), as described by Noor et al. (2023). For the training of each model, fivefold cross‐validation was used to guarantee the reliability and robustness of the model and its training process. Each model's performance was assessed through widely accepted measures, including precision, recall, *F*1 score, accuracy, and area under curve (AUC)–receiver operating characteristic (ROC). The best‐performing model was serialized in the Python programming language through the pickle module, making it easily saved and reused to predict new data without the necessity to retrain the model. This process is known as model serialization. This serialized model predicted activity on a new data set containing 19,000 polyphenol compounds. To prioritize drug‐like compounds, compounds were screened by Lipinski's Rule of Five (Ivanović et al. 2020), which identifies compounds that are likely to have oral bioavailability in humans.

### 2.9 Molecular Docking Study

Once active compounds had been identified through machine learning techniques, molecular docking studies were done to analyze the compounds' interactions with the protein of interest. In the protein's three‐dimensional structure, we removed bound ligands and water molecules, along with the addition of polar hydrogen atoms. Docking simulations were performed with PyRx, a graphical user interface for AutoDock Vina (Dallakyan and Olson 2015). The docking grid was centered on the active site of the protein, and the compounds were ranked according to their binding affinity calculated. Those compounds that were further analyzed had a binding energy of at least –7.00 kcal/mol, indicating a strong interaction. Among the top‐scoring compounds in the different classes of chemical compounds, the target protein was identified as an improved option. This reveals the potential of the site for subsequent drug design.

### 2.10 MD Simulation

MD simulations were performed using the GROMACS (Groningen Machine for Chemical Simulations) software program (v.2023.5) (Hess et al. 2011). The simple point charge/extended water system was used to solvate the system, and the protein was positioned 0 nm from the box border. To achieve a physiological ion concentration of 0 and to stabilize the system. Energy minimization to remove any steric clashes or poorly formed connectivities was done using the steepest descent algorithm. Thereafter, the equilibration process was done in two steps. Initially, NVT equilibration was performed for 100 ps at 300 K using the V‐rescale thermostat. Thereafter, the Number of particles – Pressure – Temperature (constant) (NPT) equilibrium was performed also for 100 ps with the Parrinello‐Rahman barostat at 1 bar. During the production stage, each complex was subjected to 100 ns of MD simulation at 300 K and 1 bar using the NPT ensemble for the specified folding stage and for equilibrated conditions. Then, the coordinates were saved every two ps, the time step was 2 fs, and all bond lengths were constrained using the library of integrated network‐based cellular signatures technique. Several important metrics were used in the analysis phase to characterize the behavior of the protein‐ligand complexes. The time‐dependent structural stability of each complex was evaluated using the Root Mean Square Deviation (RMSD). To evaluate the degree of flexibility of each protein residue, the Root Mean Square Fluctuation (RMSF) was calculated. To gauge the compactness of the protein structures, the radius of gyration (RoG) was calculated.

## 3 Results

### 3.1 Distribution and Prevalence of *E. coli* Isolates

A total of 350 *E. coli* isolates were obtained from pediatric patients aged 0–5 years, comprising 175 DEC and 175 non‐DEC strains. The diarrheagenic isolates were recovered from children presenting with diarrhea, while nondiarrheagenic isolates were obtained from children without diarrheal symptoms. Among the DEC strains, the most prevalent pathotype was EPEC (35%), followed by ETEC (25%), DAEC (20%), EHEC (15%), and EIEC (10%).

These findings indicate a high representation of EPEC and ETEC among diarrheagenic isolates, consistent with their established role as major etiological agents of pediatric diarrhea. Non‐DEC strains, although nonpathogenic, were included for comparative genomic and resistance profiling. The detailed distribution of isolates according to age group, clinical status, and pathotype is presented in Supporting Information File 2: Tables S1–S5 and Supporting Information File 1: Figures S1–S3.

### 3.2 Antibiotic Susceptibility Profiles of *E. coli* Isolates

Antibiotic susceptibility testing demonstrated widespread resistance among DEC strains. High resistance was observed to ampicillin (80%–97%), erythromycin (92%–100%), tetracycline (82%–95%), and trimethoprim‐sulfamethoxazole (63%–90%). Moderate resistance occurred against ciprofloxacin (42%–88%) and gentamicin (11%–77%), while carbapenems (imipenem and meropenem) and colistin remained largely effective, showing resistance rates below 20% in most pathotypes. Among DEC pathotypes, EPEC and EHEC exhibited the highest MDR patterns. These results emphasize the increasing resistance to conventional antibiotics and support the continued effectiveness of carbapenems and colistin as last‐resort options (Table 1). On the basis of Table 1, each DEC pathotype exhibited high‐level resistance to multiple antibiotic classes (EPEC and EHEC, eight classes; ETEC, seven; EIEC, six; EAEC and DAEC, five), indicating a high likelihood of widespread MDR among isolates (Figures S4 and S5).

**Table 1:** Percentage resistance for antimicrobials tested in different serotypes of *Escherichia coli*.

Group of antibiotics	Antibiotics	EPEC		EAEC		ETEC		EHEC		EIEC		DAEC	
		R	S	R	S	R	S	R	S	R	S	R	S
β‐Lactam drugs	Pencicillins Amoxicillin (AMP)	80	20	70	30	67.5	32.5	90	10	97.2	2.8	88	12
	Cephalosporins	93	7	36	64	86	14	75	25	61.7	38.2	65	35
	Piperacillin/tazobactam (TZP)	12.5	87.5	25	75	08	92	11	89	18	82	10	90
Carbapenems	Imipenem (IMI)	32	68	09	91	5	97	2.3	97.6	9.7	90.3	6	94
	Meropenem (MER)	5	95	11	89	7	93	16	84	10.7	89.3	2	98
Fluoroquinolones	Ciprofloxacin (CIP)	65	35	88	12	77.4	22.5	85.4	76.5	47	53	42	58
Aminoglycosides	Gentamicin (G)	70	30	44	56	11	89	77	23	73.1	26.9	38	62
Tetracyclines	Tetracycline (TE)	94	6	95.5	4.5	92.6	7.4	86	14	81.7	18.3	54	46
Phenicols	Chloramphenicol (C)	60	40	75.4	24.6	55	45	46	54	58	42	43	57
Sulfonamides	Trimethoprim‐sulfamethoxazole/cotrimoxazole (SXT)	63	37	46.6	53.4	84.4	15.6	89.9	10.1	22	78	59	41
Polymyxins	Colistin (CT)	15.3	84.6	32.9	67.1	32.9	94	58	42	47	53	22	88
Macrolides	Erythromycin (E)	96	4	98	2	92	8	100	0	95	5	97	3

Abbreviations: DAEC, diffusely adherent *E. coli*; EAEC, enteroaggregative *E. coli*; EHEC, enterohemorrhagic *E. coli*; EIEC, enteroinvasive *E. coli*; EPEC, enteropathogenic *E. coli*; ETEC, enterotoxigenic *E. coli*.

### 3.3 Molecular Analysis

**Figure 1:**
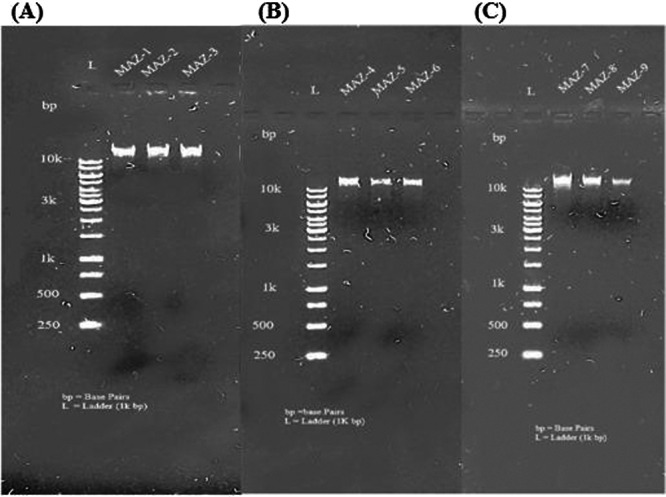
Agarose gel electrophoresis visualization of genomic extracted DNA of isolated bacterial strains. Bacterial DNA (3 µL) was loaded (Lanes 1–3); L = 1 kb‐bp DNA molecular weight ladder. (A) Lane 1 = MAZ‐1, Lane 2 = MAZ‐2, Lane 3 = MAZ‐3; (B) Lane 1 = MAZ4, Lane 2 = MAZ‐5, Lane 3 = MAZ‐6; (C) Lane 1 = MAZ‐7, Lane 2 = MAZ‐8, Lane 3 = MAZ‐9.

**Figure 2:**
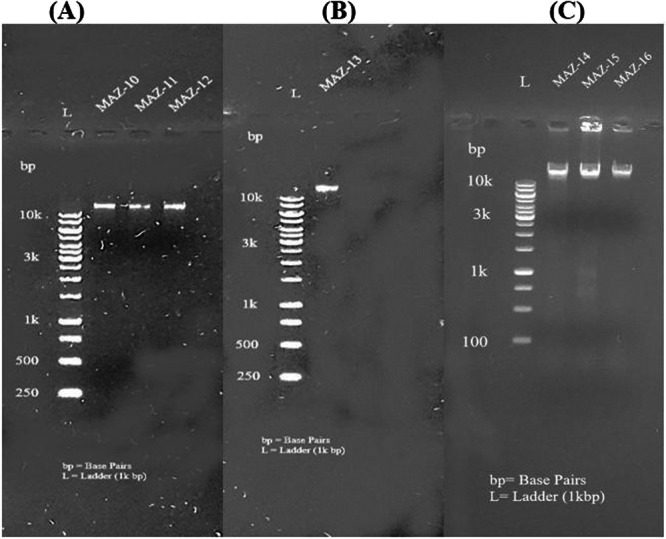
Agarose gel electrophoresis visualization of genomic extracted DNA of isolated bacterial strains. (A) Lane 1 = MAZ‐10, Lane 2 = MAZ‐11, Lane 2 = MAZ‐12; (B) Lane 4 = MAZ‐13; (C) Lane 1 = MAZ‐14, Lane 2 = MAZ‐15, Lane 3 = MAZ‐16.

The inventive extraction of DNA was demonstrated by the distinct bands that were present close to the wells, along with the absence of any stains. Figures 1 and 2 represent agarose gel electrophoresis results, run to confirm the presence of DNA in the samples labeled MAZ‐1 to MAZ‐16. The presence of distinct bands in each sample lane indicates that DNA was successfully detected in these samples. The size of the DNA fragments can be approximated by comparing them to the ladder's reference bands.

**Figure 3:**
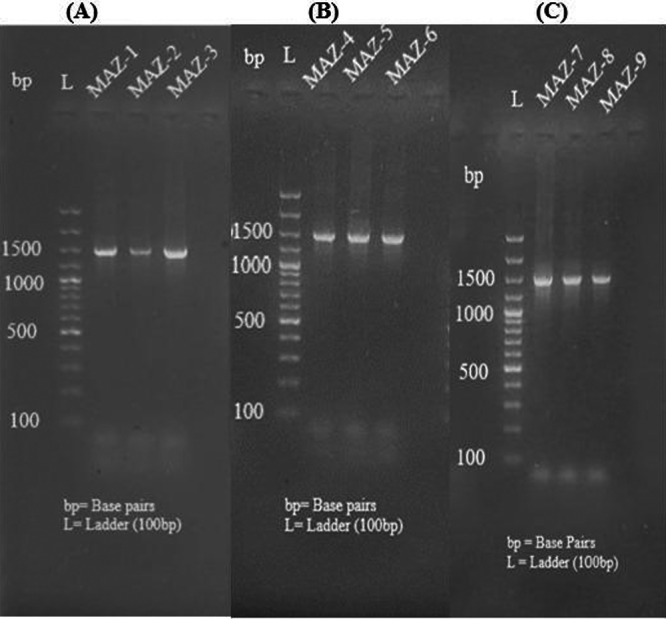
Agarose gel electrophoresis visualization of amplicon. (A) Lane 1 = MAZ‐1, Lane 2 = MAZ2, Lane 3 = MAZ‐3; (B) Lane 1 = MAZ‐4, Lane 2 = MAZ‐5, Lane 3 = MAZ‐6; (C) Lane 1 = MAZ‐7, Lane 2 = MAZ‐8, Lane 3 = MAZ‐9.

**Figure 4:**
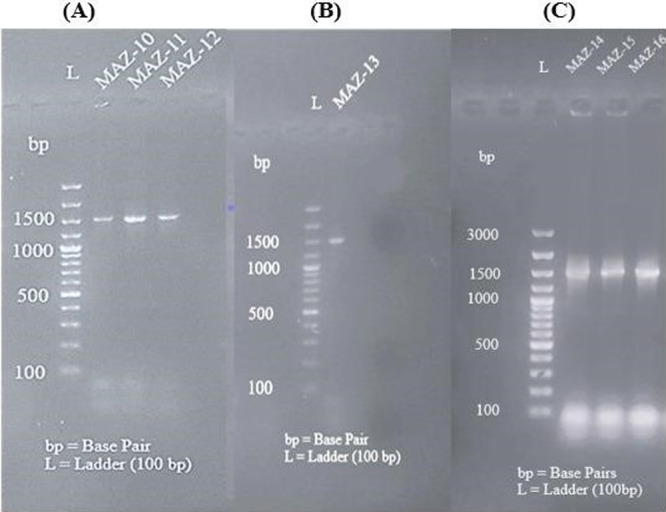
Agarose gel electrophoresis visualization of amplicon. (A) Lane 1 = MAZ‐10, Lane 2 = MAZ11, Lane 3 = MAZ‐12; (B) Lane 1 = MAZ‐13; (C) Lane 1 = 14, Lane 2 = MAZ‐15, Lane 3 = MAZ‐16.

As seen in Figures 3 and 4, the amplified DNA fragments for all distinct bacterial strains were seen on the gel with approximate sizes based on the produced amplicon after 16S rRNA gene amplification.

### 3.4 Multiplex PCR, Scheme to Identify DEC Strains

Multiplex PCR successfully identified DEC pathotypes based on virulence gene profiles. The most frequently detected virulence genes were *eae* (EPEC, 35%), *elt* and *estA* (ETEC, 25%), *stx*1 and *stx*2 (EHEC, 15%), *ipaH* (EIEC, 10%), and *daaE* (DAEC, 20%). These genes were visualized as distinct amplicons of expected sizes on agarose gel electrophoresis. Detection of these genes confirmed the genetic diversity and pathogenic potential of circulating DEC strains in pediatric patients. This assay allowed precise discrimination between DEC and non‐DEC strains, providing essential data for epidemiological monitoring.

### 3.5 *E. coli* species identification by DNA sequencing

Species‐level identification was confirmed through 16S rRNA sequencing, which matched reference *E. coli* sequences in the NCBI database, validating the phenotypic results.

### 3.6 Multiple Sequence Alignment for Comparison

Analysis of sequences for diarrheagenic and nondiarrheagenic strains of the same species has exposed significant differences in the amino acids present in the proteins that are located in important positions, for example, GLU86 (Glutamic acid), LEU3 (Leucine), SER90 (Serine), SER92, PRO143 (Proline), THR4 (Threonine), TRP54 (Tryptophan), and PRO7. These differences could affect changes in the structural stability and pathogenicity of the proteins, and also indicate differing patterns of molecular evolution within the diarrheagenic strains of the species.

*Structure prediction and evaluation*

Different computational tools were used to assess the quality and trustworthiness of the predicted structures of the hypothetical proteins. Out of the different tools used, the structure predicted by iterative threading assembly refinement (I‐TASSER was found to be the most trustworthy based on the multiple dimensions of evaluation.

The I‐TASSER model also attained the highest ERRAT score, meaning the model's structure was most intact with the least errors in the position of atoms. This score demonstrates that the model is reliable. In addition, the Ramachandran plot analysis offered more insight into the stereochemical quality of the predicted structure: 98.1% of the residues were situated in the most favored areas. This is a considerable portion with respect to the structural accuracy and even congruent stability of the model, meaning that nearly all the amino acids had optimal torsion angles, and the model indeed had a particular conformational stability. In contrast, the quality of the models created by AlphaFold2 and SWISS‐MODEL was worse according to the same criteria. In particular, the AlphaFold2 model had a slightly lower ERRAT score in comparison to I‐TASSER and 95.3% of the residues were in the favored regions of the Ramachandran plot. While the SWISS‐MODEL prediction was also valid, it was of a lower quality with 92.7% of the residues in the favored regions, and a more mediocre ERRAT score.

In summary, although all methodologies performed well in predicting protein structures, I‐TASSER surpassed the other options, AlphaFold2 and SWISS‐MODEL, in terms of precision and stereochemical quality. For these reasons, I‐TASSER was recognized as the most appropriate tool to use for high‐confidence structure prediction in this study.

### 3.7 Machine Learning–Based Virtual Screening

#### 3.7.1 Model Training

**Figure 5:**
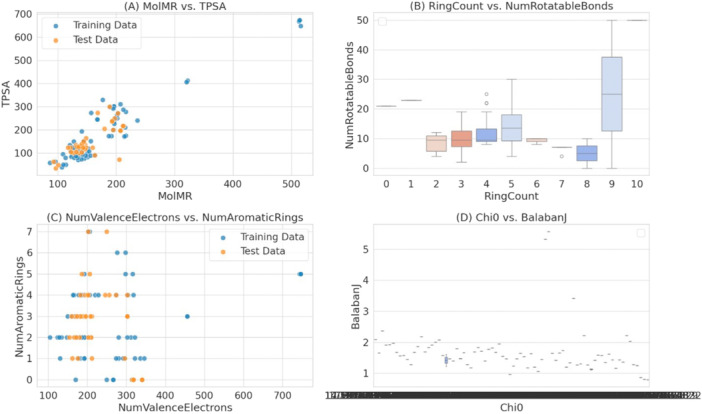
(A) Scatter plot showing the relationship between MolMR and TPSA for training and test data. (B) Box plot showing the distribution of num rotatable bonds against ring count for the data set. (C) Scatter plot showing the relationship between NumValenceElectrons and NumAromaticRings for training and test data. (D) Scatter plot of Chi0 versus BalabanJ. TPSA, topological polar surface area.

A set of 132 small molecules and decoys linked to hypothetical proteins was assembled for the purpose of machine learning model training. For model development, essential chemical features for each small molecule were calculated using Roger Dimick's Kit. The data set was divided to include a separate training set, testing set, and an external test set for generalization evaluation. Most of the compounds are concentrated near the lower values for both descriptors of the MolMR and topological polar surface area scatter plot, with a few outliers at higher values, as illustrated in Figure 5A. The plot in Figure 5B demonstrates the distribution of NumRotatableBonds with respect to RingCounts, where the structure variability is attributed to the higher counts of the rings (especially 9 and 10) for the broader range of rotatable bonds. The comparison of NumValenceElectrons and NumAromaticRings in Figure 5C reflects that the majority of the molecules contain 0–4 aromatic rings. The range of the valence electrons within the data set is rather broad, while the espoused limit of the aromatic rings is also broad. In Figure 5D, the plotting of Chi0 against BalabanJ demonstrates that the general majority of molecules are lower in both descriptors, although some molecules are higher and deviate significantly. The significance of these plots lies in the chemical diversity present in the set, critical for developing effective and generalizable machine learning models for virtual screening.

#### 3.7.2 Model Evaluation

**Figure 6:**
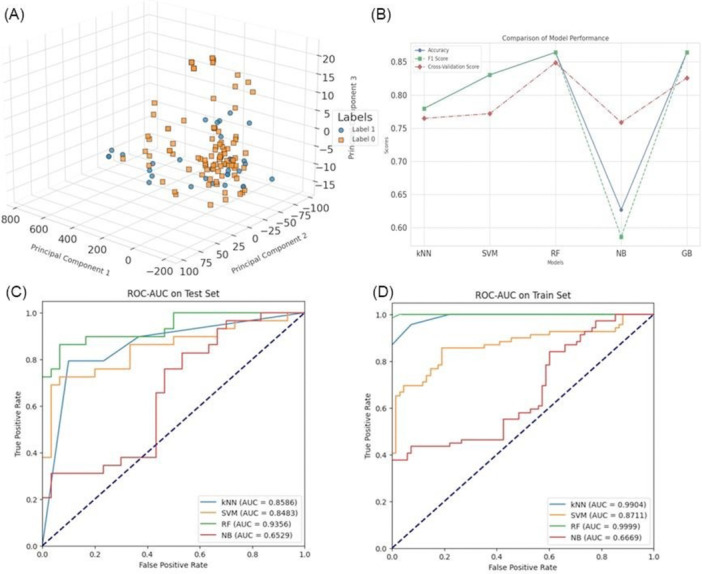
(A) Three‐dimensional PCA plot of the first three principal components, colored by labels, for the data set. (B) Line plot comparing model performance metrics (accuracy, *F*1 score, and cross‐validation score) across five models: kNN, SVM, RF, NB, and GB. (C) ROC curves for the test set, illustrating how various models performed. (D) ROC curves illustrating the training set's performance for several models. AUC, area under curve; GB, gradient boosting; kNN, *k*‐nearest neighbor; NB, Naive Bayes; PCA, principal component analysis; RF, random forest; ROC, receiver operating characteristic; SVM, support vector machine.

Several machine learning models were trained and evaluated on the data set following feature extraction and data set preparation. kNN, SVM, RF, NB, and Gradient Boosting (GB) are these. With a high accuracy and an *F*1 score of about 0.85, RF is found to have the highest overall performance when comparing all of the aforementioned models based on the major assessment factors (Figure 6) On the other hand, the NB model was much worse, having notably lower accuracy, *F*1 coefficient and cross‐validation coefficient compared with all the models might because of its incapability to elaborate correlation and interaction in the data set. Thus, the ROC–AUC scores were utilized to evaluate the performance of the models further. Furthermore, the highest AUC value indicates that the RF was the best model distinguishing between the two classes, as confirmed by the statistically significant differences between the models based on AUC values in the test set in the kNN and SVM models as well. NB was much lower in AUC, indicating the poor classification capability of the algorithm. When evaluated on the training set, both RF and kNN achieved AUCs very close to 1, which indicates overfitting. SVM produced reasonable performance, which showed a good generalized ability on both the training set and the testing set. However, GB seemed to overfit, as its performance was substantially worse in the test set in comparison to the training set. Adding up, the findings show that RF is the best model to use with a learning rate of 0.01 to avoid overfitting. In contrast, NB failed to identify the patterns in the data distribution, while GB performance, while not as poor as NB, fluctuated between the training and testing data sets further underlining the need for proper model calibration on this higher‐order data set.

### 3.8 Screening of Phenolic Compounds With Drug‐Like Potential Compounds With Drug‐Like Potential

**Figure 7:**
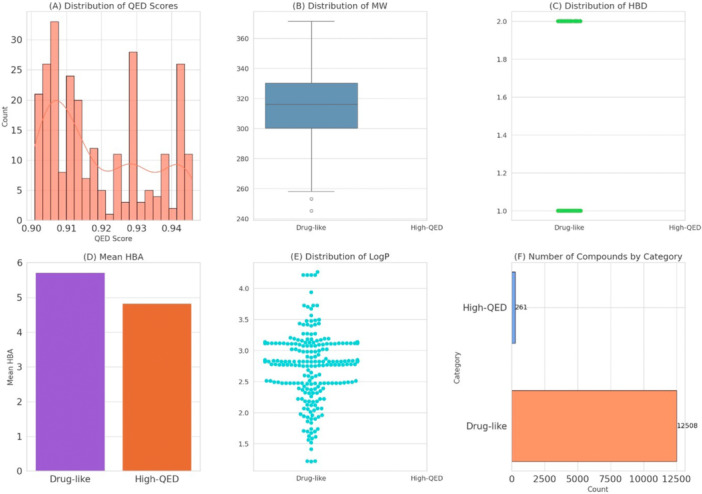
(A) Histogram showing the distribution of QED scores, (B) box plot showing the distribution of molecular weight (MW) across drug‐like and high‐QED categories, (C) strip plot showing the distribution of HBD across drug‐like and high‐QED categories, (D) bar plot showing the mean HBA across drug‐like and high‐QED categories, (E) swarm plot showing the distribution of Log *P* across drug‐like and high‐QED categories, and (F) bar plot showing the number of compounds in the drug‐like and high‐QED categories. HBA, hydrogen bond acceptor; HBD, hydrogen bond donor; QED, quantitative estimate of drug.

Using the trained RF model, a library of 989 phenolic compounds was screened for drug‐like potential. On the basis of the RF model's predictions, 12,509 compounds were identified as drug‐like candidates (Figure 7). To further refine the selection, the drug‐likeness of these phenolic compounds was assessed using their Quantitative Estimate of Drug‐likeness (QED) scores. Of these, only 261 compounds were identified as promising candidates with a QED score greater than 0.90. From this subset of 261 compounds, 245 were determined to be nontoxic and were selected for further analysis.

### 3.9 Molecular Docking

**Figure 8:**
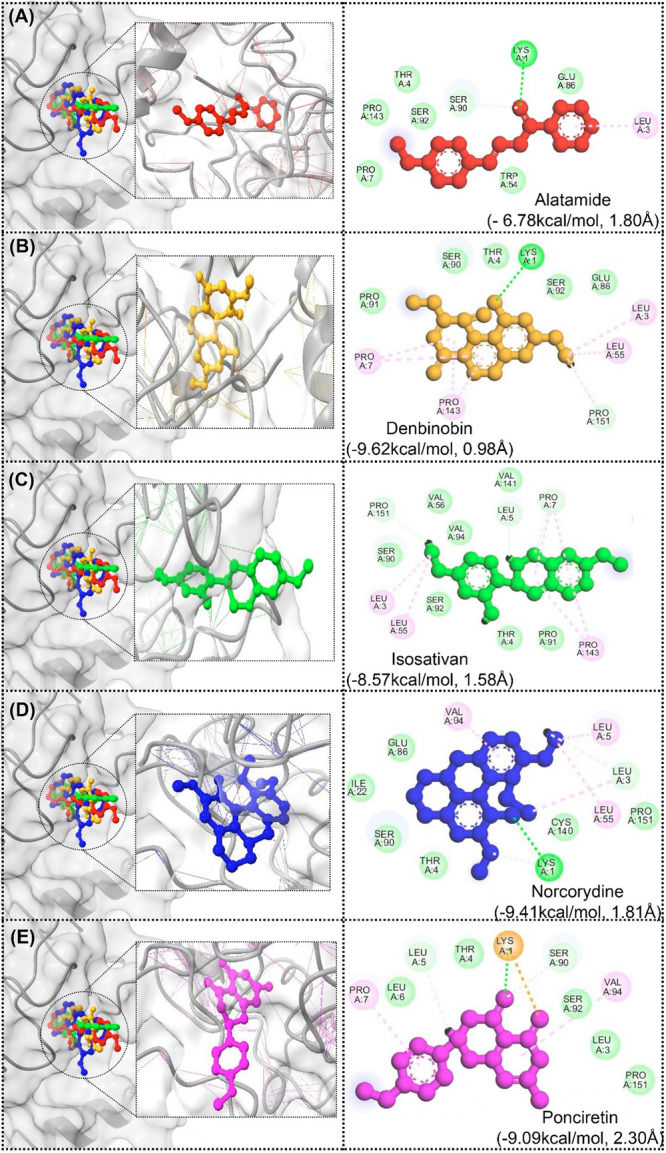
Molecular docking of various compounds with a hypothetical protein structure. (A) Alatamide (−6.78 kcal/mol, 1.80 Å): The binding interactions of alatamide are shown in three‐dimensional (3D) (left) and 2D (right), highlighting key residues involved in the interaction (e.g., PRO, SER, and LYS). (B) Denbinobin (9.62 kcal/mol, 0.98 Å): This panel shows the docking of denbinobin, demonstrating tighter binding energy and shorter binding distance compared with alatamide. (C) Interaction analysis of Isosativan with protein (D) Norcorydine (−9.41 kcal/mol, 1.81 Å): Docking results for norcorydine are shown, with interactions involving multiple residues. (E) Poncretin (−9.09 kcal/mol, 2.30 Å).

The outcomes from the molecular docking studies indicate that the polyphenolic compounds that were analyzed are predicted to interact quite well with the target proteins, as shown in Figure 8 with respect to the negative binding affinity values in kcal/mol. With respect to binding interactions in molecular docking, most negative binding affinities are indicative of stronger interactions between the ligand and the proteins. In this instance, the strongest binding affinity was predicted for Denbinobin at –9.62831 kcal/mol; this was followed by Norcorydine at –9.4144 kcal/mol, and Ponciretin at –9.09486 kcal/mol. Therefore, these three compounds are predicted with a higher likelihood to effectively target the proteins. Moreover, the conformational stability of the binding poses was predicted using RMSD values. Denbinobin had a low RMSD of 0.983739, which predicted a conformational stability of the bind. On the contrary, a higher RMSD of 2.302617 for Ponciretin means that the predicted binding with the target protein was less stable in a dynamic sense.

In conclusion, the results from molecular docking offer evidence for Denbinobin and Norcorydine to be the most advanced compounds in the study, as docking posed the strongest conformations with high binding affinity and stability. Thus, these compounds should be the focus of the following studies in validation of binding affinity and in the study of interaction with target proteins for their possible therapeutic value.

### 3.10 MD Simulations

**Figure 9:**
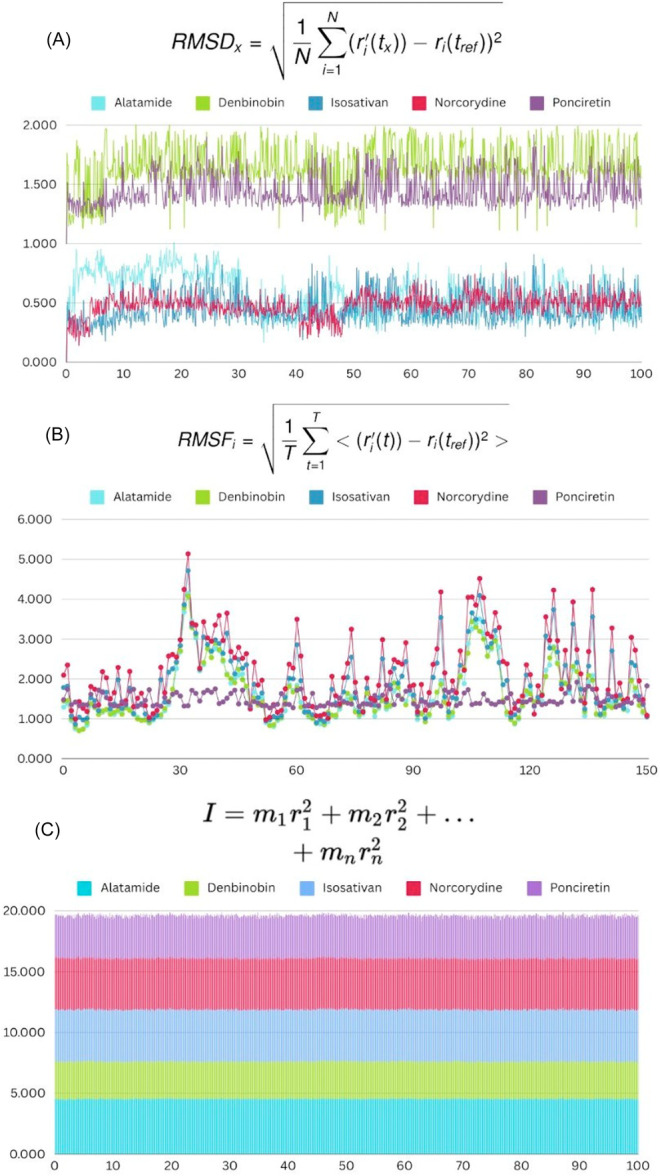
Analysis of structural stability, flexibility, and rotational behavior of five compounds. (A) Root mean square deviation (RMSD) values over time for alatamide, denbinobin, isosativan, norcorydine, and poncretin, indicating overall structural stability. (B) Root mean square fluctuation (RMSF) showing the flexibility of different regions within the compounds. (C) Radius of gyration.

The next approach to fully characterize the affinity of the compounds to the proteins is the MDs simulations. An in‐depth analysis of the results for the five compounds, Alatamide, Denbinobin, Isosativan, Norcorydine, and Poncretin, can be performed in terms of their structural behavior and stability using RMSD, RMSF, and RoG (Figure 9). Regarding the stability of the EPEC, which was the most common pathogen, found compounds (Figure 9A), the RMSD values over time convey an understanding of the overall stability exhibited by the compounds. For instance, Alatamide and Isosativan possess lower RMSD values, indicating greater stability in those respective compounds for the duration of the simulations. On the other hand, Denbinobin and Poncretin seemed to emanate large structural shifts or fluctuations, hence indicating instability with higher RMSD values. Norcorydine appears to have an intermediate stability since the RMSD values maintain a relative distance between the observed extremities of the other two compounds.

In Figure 9B, the analysis of the results focuses on the prediction of the RMSF, in which the flexibility of the various regions of the compound is described, and structural regions could be construed as rigid. Norcorydine is interesting in that it has a number of regions with flexibility as described by the peaks in the RMSF. Alatamide and Isosativan once again demonstrate more substantially rigid structures as described by the low fluctuations observed. Denbinobin and Poncretin exhibited the described moderate fluctuations between their teleporting rigid zones with weaker structures that are more flexible to minor fluctuations in balance with the rest of their stronger frameworks.

For the five compounds described above, Figure 9C displays the RoG, which in turn indicates the compactness of each molecular structure as an overall possessed value. Results indicate that Poncretin has the largest RoG, indicative of a more extended and less compact structure. Norcorydine and Denbinobin, on the other hand, have moderate radii of gyration, which indicates a more neutral balance between extension and compactness. Alatamide and Isosativan possess the smallest radii of gyration, signifying that they are the most compact of the group. This suggests that there are important differences in the compactness of these structures, and the varying degrees of compactness of these compounds may have biological implications on the binding and affinity interactions of each compound with proteins. Alatamide and Isosativan, in particular, are more likely to act against DEC bacteria, as they are more compact and stable based on their structural properties. Their compact and stable structures suggest that targeting key proteins responsible for the pathogenicity of these bacteria might result in the development of viable therapeutics.

## 4 Discussion

Diarrheal infections, especially in children, continue to be a major public health concern, with *E. coli* contributing to both enteric and extra‐enteric infections. *E. coli* can be a gut commensal and also acquire virulent factors that make it a pathogen. This study is the first in the region to describe the molecular features, including the antibiotic resistance patterns of both DEC and non‐DEC strains, and the need for epidemiological surveillance with enforceable treatment. These results also document *E. coli* as a causal agent for 30%–40% of diarrhea worldwide, a trend that is consistent with prior research (Fellenz et al. 2024). The prevalence of DEC in this study (10%–35%) is comparable to other research conducted in lower‐income countries, although some localized outbreaks in the country of Pakistan have a prevalence of up to 65% (Masood et al. 2022). Of the 350 isolates reviewed, half were clinically identified as DEC strains. The age‐specific data and analysis showed children 3–5 years of age had the greatest prevalence of *E. coli*‐associated diarrhea, and this confirmed the expected epidemiological patterns (Jesser and Levy 2020).

The distribution and resistance patterns of *E. coli* strains were also influenced by patient demographics. The higher prevalence of diarrheagenic strains among children aged 3–5 years may reflect increased environmental exposure, underdeveloped immunity, and poor hygiene practices typical of this age group. No significant difference was observed between male and female patients. Underlying health conditions, such as mild malnutrition, were noted in some diarrheal cases, which may have predisposed these children to infection and increased antibiotic resistance risk.

In 35% of diarrheagenic cases, EPEC was the most common pathogen. The high prevalence of the eae gene, which encodes the adhesion factor of EPEC, further substantiates the link between EPEC and severe, protracted diarrhea in young children. EPEC was identified in 5% of cases that did not have diarrhea, which suggests possible asymptomatic carriage. This supports the findings of Dias et al. (2016), which stated that EPEC continues to dominate as a pathogen in DEC and its presence is ubiquitous in developing countries. The next most prevalent DEC pathotype, ETEC, was present in 25% of diarrheagenic cases and was associated with the estA and eltB genes. This correlates with the global picture of ETEC, which disproportionately affects young children in endemic areas and continues to be a leading cause of travel‐related diarrhea. EHEC, which produces Shiga toxins and is particularly dangerous, was found in 15% of cases. The low prevalence of EIEC (10%) in both symptomatic and asymptomatic cases could suggest that EIEC is a pathogen that goes unnoticed. In contrast, DAEC was present in 20% of both groups, which could indicate its potentially latent pathogenicity (Yoon et al. 2024).

The study shows that AMR poses an increasingly serious threat to public health, given that resistance to the more common classes of antibiotics continues to increase. The highest levels of recorded resistance to ampicillin and other penicillins and β‐lactam antibiotics. The findings of the study show that the older antibiotics, especially sulfonamides, and trimethoprim‐sulfamethoxazole in particular (75.5%)—still present a challenge in treating DEC. The global increasing trend of antibiotic resistance, especially in South Asia, is captured by the DEC strains resistance to macrolides, especially ciprofloxacin (45.5%) (Zhou et al. 2022). The 100% resistance of DEC strains to erythromycin is alarming and a clear indication that novel treatment alternatives are warranted. That the carbapenems imipenem and meropenem also had 25% resistance suggests that the carbapenems still hold value as last‐resort treatment options. There is inadequate control of AMR, especially in regions that have a high burden of diarrheal disease (Denamur et al. 2021).

Although this study did not include molecular screening of antibiotic resistance genes, the observed resistance patterns highlight the need for future genomic investigations to identify the underlying resistance determinants and mobile genetic elements contributing to MDR in *E. coli*. Also, plasmid carriage and clonal relationship analyses were not conducted; future studies incorporating plasmid profiling and molecular typing will be essential to elucidate the genetic mechanisms and potential transmission routes of resistant *E. coli* strains. In resource‐limited settings like Pakistan, continuous AMR surveillance can be integrated into public health strategies by utilizing existing hospital microbiology laboratories as sentinel sites. Routine diagnostic data, such as that generated in this study, can be shared with national surveillance networks to monitor emerging resistance trends. Gradual inclusion of molecular analyses through academic–clinical collaborations would strengthen sustainable AMR tracking and antibiotic stewardship efforts.

Molecular characterization using 16S rRNA gene sequencing identified each isolate's specific genetic identity and confirmed the presence of virulence traits in the DEC strains. Phylogenetic analysis using the Neighbor‐Joining method indicated that DEC strains are closely phylogenetically related and clustered within the Escherichia genus. Moreover, DEC strains are distinct and separated from non‐DEC strains, and from outgroup species, such as *Pseudomonas aeruginosa* and *Salmonella typhi*. This indicates the genetic closely relatedness of the DEC strains and divergence in evolution. Analysis of the DEC functional traits performed using phylogenetic investigation of communities by reconstruction of unobserved states (PICRUSt) showed that DEC strains have increased metabolism and immune system interaction compared with non‐DEC strains, which indicates that they are adapted to host colonization and have enhanced survival during diarrheal disease. Performing hierarchical clustering showed that pathways related to immune response and infectious disease were more enhanced in DEC strains, while pathways related to genetic information processing were more enhanced in non‐DEC strains. These findings demonstrate that DEC and non‐DEC strains have core differences in their functional and metabolic profiles (Zhang et al. 2021).

PICRUSt‐based functional pathway analysis revealed distinct differences between DEC and non‐DEC strains, offering insight into their host–pathogen interactions and potential therapeutic implications. Diarrheagenic strains exhibited higher predicted activity in metabolic and human disease‐associated pathways, particularly those related to infectious and immune system diseases, such as epithelial cell signaling, bacterial invasion of epithelial cells, and *Staphylococcus aureus* and *Shigella* infections. Enhanced functions related to the immune system and environmental adaptation further suggest a stronger ability of DEC strains to colonize and persist in the host intestinal environment. In contrast, nondiarrheagenic isolates showed higher enrichment in genetic information processing pathways, including replication and repair, indicating a more commensal‐like or stable adaptation to the gut environment. These functional distinctions underscore the metabolic flexibility and immune evasion potential of DEC strains, highlighting pathways that could be explored as novel therapeutic or preventive targets against DEC.

In assessing potential new therapeutic candidates, the authors utilized machine learning and docking methods to find promising candidates to target compounds used to treat *E. coli*‐associated diarrhea. Using the NDSS platform, the authors focused attention on polyphenolic compounds Denbinobin and Norcorydine, citing strong promising candidates and stable interaction and attachment to *E. coli* target proteins, which was supported by low RMSD values. More positive results came when the authors ran MD simulations and confirmed prior results on the stability of attachment to *E. coli* and built upon those results to carry Isosativan and Alatamide, which has strong therapeutic potential for DEC infections. These compounds merited further attention as they demonstrated strong therapeutic potential. This study responds to ongoing efforts to counteract AMR and broaden the scope to include *E. coli*‐related diarrheal diseases (Jiang et al. 2022).

Literature searches revealed no prior in vitro or in vivo data demonstrating anti‐*E. coli* activity for Alatamide or Isosativan. Alatamide's enamide scaffold and propolis‐derived isosativan have been linked to antimicrobial activity in related contexts (notably antifungal for isosativan), but *E. coli* MICs have not been reported. Accordingly, our hits are predictions that require experimental validation against DEC strain.

## 5 Conclusion

This study offers a detailed examination of the distribution, molecular features, and AMR of DEC and non‐DEC strains from children, especially focusing on the pediatric demographic. EPEC and ETEC were the most identified pathotypes, affirming most of the role of DEC in causing diarrhea in children. The finding of the eae gene in EPEC demonstrates a close association with causing diarrheal disease, while the enterotoxigenic properties of the ETEC reinforces the role of ETEC in causing secretory diarrhea. The presence EHEC, EIEC, and DAEC in symptomatic and asymptomatic cases speaks to the different pathogenic mechanisms of the *E. coli* strains. The study also noted concerning levels of resistance to first‐line antibiotics, especially ampicillin, trimethoprim‐sulfamethoxazole, and erythromycin, signaling the need for a clear antimicrobial stewardship and resistance management. The remaining efficacy of carbapenems and colistin demonstrates their ongoing importance as last‐line agents. Findings from DNA and phylogenetic analyses indicated separate evolutionary development of DEC and non‐DEC strains, while pathway analysis showed DEC strains with advanced metabolic and immune functions relative to non‐DEC strains. Through computational drug discovery and molecular epidemiology, Alatamide and Isosativan have been identified as candidates with good and stable binding affinities against the virulence targets of DEC. This further highlights the promise of bioinformatics and machine learning techniques in the development of new antidiarrheal therapies. In summary, this work highlights the urgent need for active surveillance for AMR, the improvement of diagnostic tools, the rational use of antibiotics, and the need for in vitro testing of the drugs identified through computational methods to manage *E. coli*‐associated diarrhea and the burden of MDR infections in children.

## Author Contributions

**Ayesha Masood:** methodology, writing – original draft. **Fatima Noor:** methodology, validation, writing – review and editing. **Abdu Rehman:** methodology. **Mohsin Gulzar Barq:** validation, formal analysis. **Shazia Iqbal:** methodology, visualization. **Muhammad Qasim Ali:** methodology, writing – original draft. **Shahzad Ahmad:** supervision, review the draft. **Syed Zeeshan Haider Naqvi:** supervision.

## Ethics Statement

The authors have nothing to report.

## Conflicts of Interest

None declared.

## Supporting information


**Figure S1:** Percentage ratio between male and female patients in different age groups. **Figure S2:** Microorganisms isolated from stool and rectal swab cultures according to age groups in diarrheal infections. **Figure S3:** Clinical features associated with DEC‐positive patients. **Figure S4:** Graphical representation of resistance for antibiotics tested in different pathotypes of *E. coli*. **Figure S5:** Graphical representation of difference in antibiotic resistant in DEC and non‐DEC.

**Table S1:** Prevalence of infectious growth in different age groups. **Table S2:** Demographics of respondents. **Table S3:** The organism positive percentage ratio between male and female patients in different age groups. **Table S4:** Distribution of different sources of specimens in different age groups. **Table S5:** Distribution of *E. coli* pathotypes in diarrheagenic and nondiarrheagenic groups.
