# Supplementary material for: Genomic Epidemiology and Machine Learning–Based Drug Discovery for Antimicrobial Resistant Diarrheagenic Escherichia coli
Source: Microbiologyopen. 2026 Feb 22;15(1):e70236. doi: 10.1002/mbo3.70236 (PMC12927950; doi:10.1002/mbo3.70236)
Supplement: Supplementary file 1 — Figure S1: Percentage ratio between male and female patients in different age groups. Figure S2: Microorganisms isolated from stool and rectal swab cultures according to age groups in diarrheal infections. Figure S3: Clinical features associated with DEC‐positive patients. Figure S4: Graphical representation of resistance for antibiotics tested in different pathotypes of E. coli. Figure S5: Graphical representation of difference in antibiotic resistant in DEC and non‐DEC. [file MBO3-15-e70236-s001.pdf]

Supplementary File 1

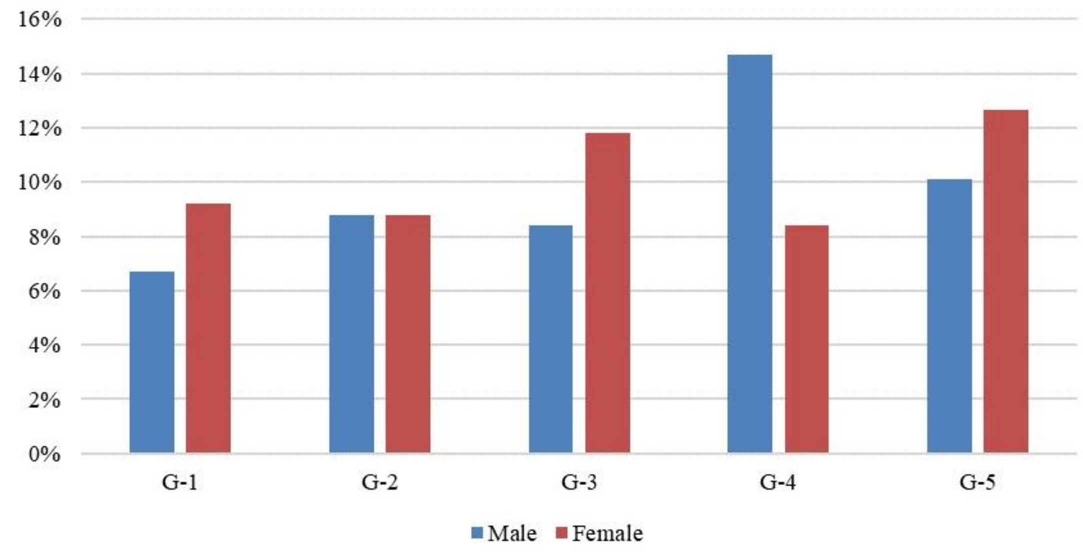

Figure S1: Percentage ratio between male and female patients in different age groups

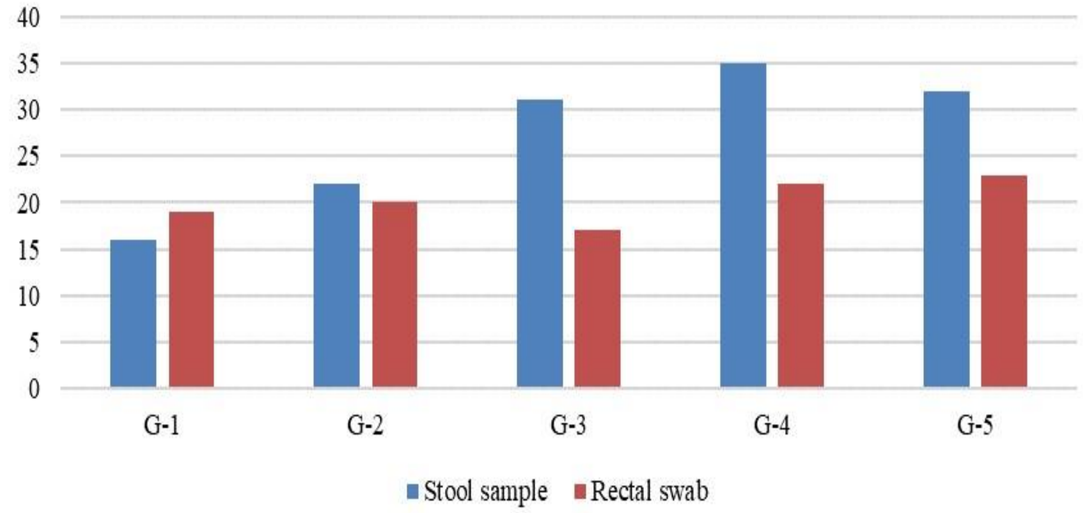

Figure S2: Microorganisms isolated from stool and rectal swab cultures according to age groups in diarrheal infections.

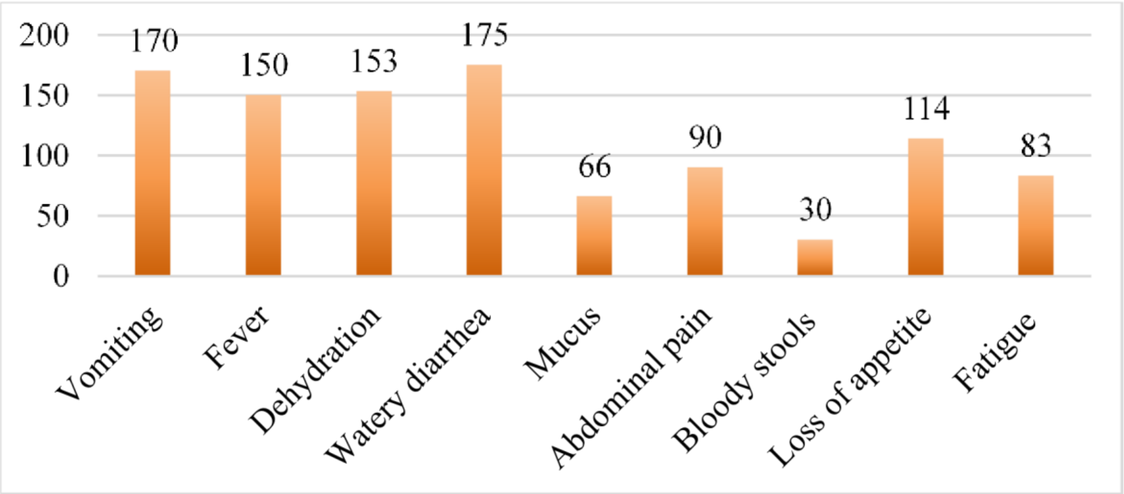

Figure S3: Clinical features associated with DEC-positive patients

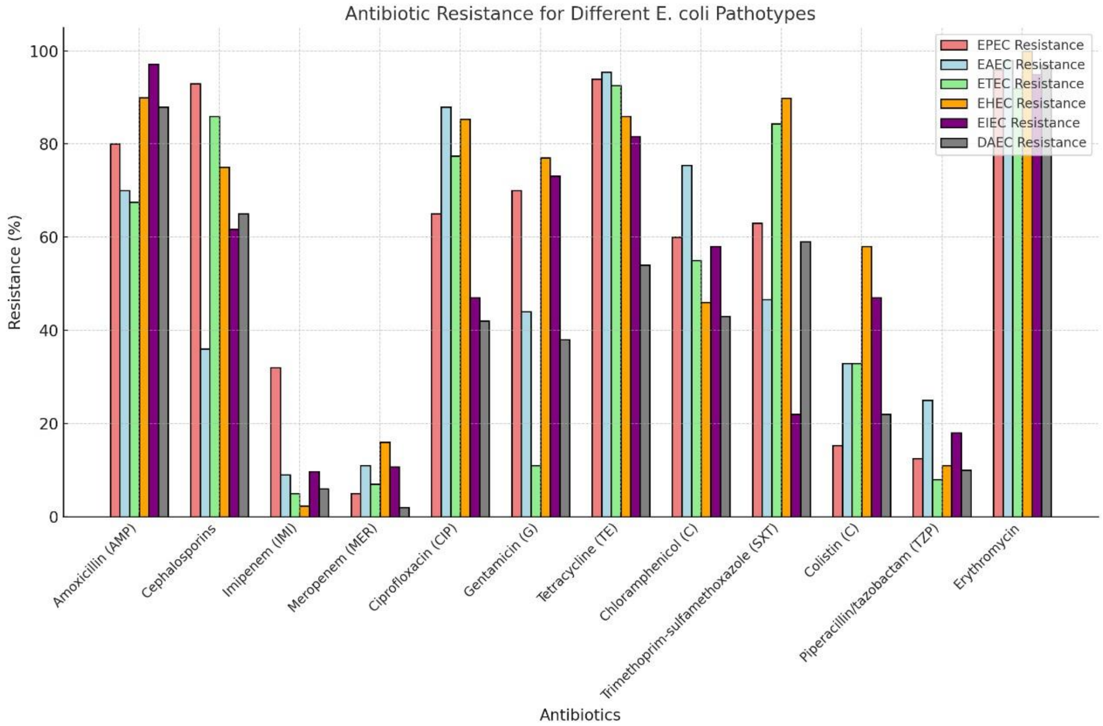

Figure S4: Graphical representation of resistance for antibiotics tested in different pathotypes of *E. coli*

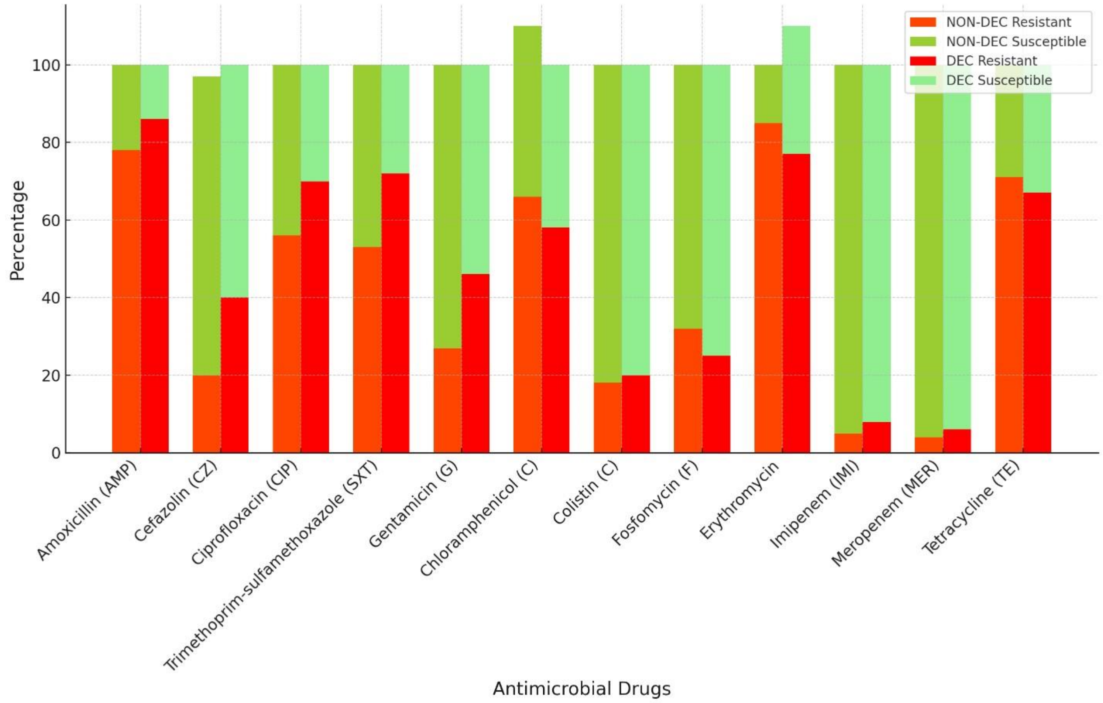

Figure S5: Graphical representation of difference in antibiotic resistant in DEC and non-DEC
