# Supplementary material for: Genomic Epidemiology and Machine Learning–Based Drug Discovery for Antimicrobial Resistant Diarrheagenic Escherichia coli
Source: Microbiologyopen. 2026 Feb 22;15(1):e70236. doi: 10.1002/mbo3.70236 (PMC12927950; doi:10.1002/mbo3.70236)
Supplement: Supplementary file 2 — Table S1: Prevalence of infectious growth in different age groups. Table S2: Demographics of respondents. Table S3: The organism positive percentage ratio between male and female patients in different age groups. Table S4: Distribution of different sources of specimens in different age groups. Table S5: Distribution of E. coli pathotypes in diarrheagenic and nondiarrheagenic groups. [file MBO3-15-e70236-s002.docx]

**Supplementary File 2**

**Table S1:** Prevalence of infectious growth in different age groups

| Groups | Age (years) | Samples size (n) | Growth (%) |
| --- | --- | --- | --- |
| G-1 | 1 day – 1 year | 70 | 24 (34%) |
| G-2 | 1 year – 2 years | 70 | 20 (29%) |
| G-3 | 2 years – 3 years | 70 | 30 (43%) |
| G-4 | 3 years – 4 years | 70 | 35 (50%) |
| G-5 | 4 years – 5 years | 70 | 37 (53%) |

**Table S2:** Demographics of respondents

|  |  | **Frequency N= 350** | **Percentage %** |
| --- | --- | --- | --- |
| **Gender** | Male | 163 | 48.95 |
|  | Female | 187 | 51.05 |
| **Groups** | A – Diarrheagenic | 175 | 50 |
|  | B - Non-Diarrheagenic | 175 | 50 |

**Table S3:** The organism positive percentage ratio between male and female patients in different age groups

| **Groups** | **Age (years)** | **Male (n)** | **Female (n)** | **Samples (n)** |
| --- | --- | --- | --- | --- |
| G-1 | 1 day-1 year | 29 (6.7%) | 41 (9.2%) | 70 |
| G-2 | 1-2 years | 35 (8.8%) | 35 (8.8%) | 70 |
| G-3 | 2-3 yeas | 28 (8.4%) | 42 (11.8%) | 70 |
| G-4 | 3-4 years | 40 (14.7%) | 30 (8.4%) | 70 |
| G-5 | 4-5 years | 31 (10.1%) | 39 (12.65%) | 70 |
| Total |  | 163 (48.95%) | 187 (51.05%) | 350 |

**Table S4:** Distribution of different sources of specimens in different age groups.

| **Groups** | **Age** | **Stool samples** | **Rectal swab** | **Total (n)** |
| --- | --- | --- | --- | --- |
| G-1 | 1day-1yrs | 31 | 39 | 70 |
| G-2 | 1-2yrs | 37 | 33 | 70 |
| G-3 | 2-3yrs | 42 | 28 | 70 |
| G-4 | 3-4yrs | 47 | 23 | 70 |
| G-5 | 4-5yrs | 45 | 25 | 70 |
| **Total** |  | 202 | 148 | 350 |

**Table S5:** Distribution of *E. coli* pathotypes in diarrheagenic and non-diarrheagenic groups

| ***E. coli* Strains** | **Percentage of DEC** | **Percentage of non-DEC** | **P value** |
| --- | --- | --- | --- |
| Enteropathogenic *E. coli* (EPEC) | 35 | 5 | < 0.01 |
| Enterotoxigenic *E. coli* (ETEC) | 25 | 5 | <0.05 |
| Enterohemorrhagic *E. coli* (EHEC) | 15 | 5 | <0.05 |
| Enteroinvasive *E. coli* (EIEC) | 10 | 7 | <0.05 |
| Diffusely Adherent *E. coli* (DAEC) | 10 | 10 | >0.05 |
